# What are the threats from antimicrobial resistance for maternity units in low- and middle- income countries?

**DOI:** 10.3402/gha.v9.33381

**Published:** 2016-09-16

**Authors:** Wendy J. Graham, Emma Morrison, Stephanie Dancer, Kaosar Afsana, Alex Aulakh, Oona M. R. Campbell, Suzanne Cross, Ryan Ellis, Siyoum Enkubahiri, Bazezew Fekad, Giorgia Gon, Patrick Idoko, Jolene Moore, Deepak Saxena, Yael Velleman, Susannah Woodd

**Affiliations:** 1 Department of Infectious Disease Epidemiology, London School of Hygiene and Tropical Medicine, London, UK; 2 The Soapbox Collaborative, Aberdeen, UK; 3 NHS Lanarkshire and Edinburgh Napier University, Edinburgh, UK; 4 Health Nutrition & Population Programme, BRAC, Dhaka Division, Dhaka, Bangladesh; 5 Northwick Park Hospital, London North West Healthcare Trust, London, UK; 6 Felege Hiwot Referral Hospital, Bahir Dar, Ethiopia; 7 School of Medical and Allied Health Sciences, University of The Gambia, Banjul, The Gambia; 8 Institute of Education for Medical and Dental Sciences, University of Aberdeen, Aberdeen, UK; 9 Indian Institute of Public Health, Gandhinagar, India; 10 WaterAid, London, UK

Global attention towards antimicrobial resistance (AMR) and the threat it presents to current and future human health has soared in the last 2 years (1, 2). A clear marker of this awakening is the presence of AMR as a priority topic at the 71st United Nations General Assembly (UNGA) in late September 2016. This high-level forum is the first to be held in the post-Millennium Development Goal (MDG) era, and its agenda reflects the 17 new Sustainable Development Goals (SDGs). The challenge of AMR is directly relevant to Goal 3 ‘Good health and well-being’, but can also be related to Goal 12 ‘Responsible consumption and production’ and Goal 6 ‘Clean water and sanitation’. The prominence of AMR at the 71st UNGA is thus not surprising. What is surprising is the comparative neglect of threats from AMR to women and children in low- and middle-income countries (LMICs) and, specifically, for the crucial environment of maternity units. Given the UN Secretary General's much repeated call to ‘leave no one behind’ in pursuit of sustainable development by 2030 (3), this neglect is unacceptable. In our article, we call for joined-up thinking and working to address the current lack of attention, evidence, and action on the threat of AMR for maternity units. The benefits of addressing this would be felt widely, but particularly by the women who become pregnant and the newborn babies potentially at risk – estimated, respectively, as 210 million and 140 million in 2015 (4).

Sepsis accounts for around 10–15% of deaths among pregnant or recently-delivered women and among neonates: virtually all of these deaths are preventable and the vast majority occur in LMICs (4, 5). Options for tackling sepsis – both preventive and curative – have long been integrated into wider efforts to reduce maternal and neonatal mortality, as in the latest Global Strategy for Women's, Children's and Adolescents’ Health (6). A defining moment in the risk to women and babies occurs at the time of labour and delivery, and this has led to policies and programmes prioritising skilled care at delivery. Seventy-five percent of births worldwide are with skilled attendants, largely in institutions. The latest evidence on the proportion of births occurring in health facilities in LMICs reveals a marked upward trend over the last 10 years, now passing the 50% tipping-point in most settings (7) (see Fig. 1). Although the proportion varies widely between countries, and within countries in terms of geographic and socio-economic differentials, the overall increase in coverage is seen as an indicator of success of the MDG era. However, evidence of the poor care that too many women and newborn babies receive in maternity units has also been mounting.

**Fig. 1:**
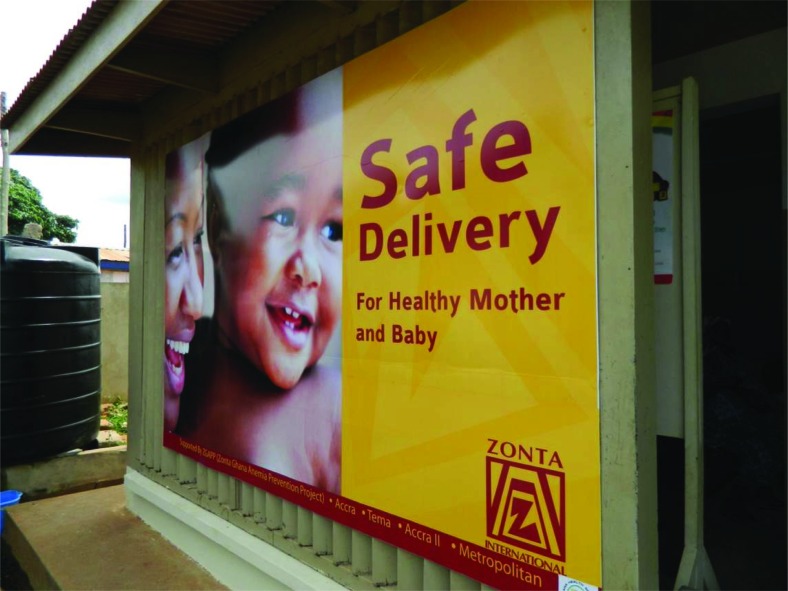
Positive signage at a maternity unit in Ghana to encourage women to attend for delivery (© 2012 Soapbox Collaborative).

The urgent need to prioritise improvements in quality of care during delivery, as well as during pregnancy, the puerperium and beyond, is one of the key messages of the call to action in the recent *Lancet* series on maternal health (8). Quality care has been defined as ‘care which is effective, safe and a good experience for the patient’ (9), and requires action on six dimensions of quality (10), including technical skills as well as infrastructure. The prevalence of healthcare-associated infections (HCAIs) reflects several of these dimensions, such as missed opportunities for prevention as well as more rational and appropriate use of antibiotics (11).

**Fig. 2:**
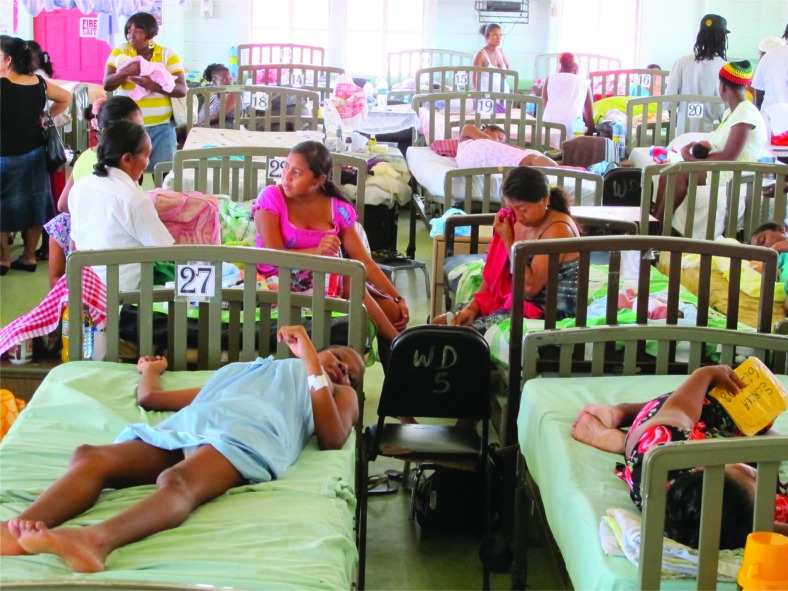
A crowded maternity unit in Guyana (© 2012 Barry Reinhart/WONDOOR Global Health Program, Courtesy of Photoshare).

The risk of maternal death from iatrogenic infections at the time of birth has been known about for centuries (12), as well as the potential for prevention through hygienic practices and birth environments. Similarly, the crucial role of antibiotics in preventing deaths from *childbed fever* (puerperal sepsis) has also been well-charted historically; for example, the contribution to the 80% decline in maternal mortality in the United Kingdom from 1935 to 1950 (13). This remarkable decline was not, of course, due solely to antibiotics but also to wider improvements in the quality of maternity services which ensured women's care experiences were indeed *effective, safe and good*. In other words, strengthened infection prevention and control (IPC) was fully integrated into quality improvement, covering enhanced practices as well as environments.

So have these historic lessons been learnt, adapted, and applied appropriately to the maternity units in LMICs that are now the location of most of the world's births? Is the full potential from primary prevention of infections at birth through clean, quality care being realised for mothers and babies? Unfortunately, evidence from a wide variety of assessments indicates a huge missed opportunity. A WHO survey (14) across 54 LMICs revealed that 38% of healthcare facilities did not have access to basic water sources and 19% to basic sanitation infrastructure. A recent detailed analysis of data from the Demographic and Health Surveys and the Service Provision Assessment for maternity units across four East African countries found that less than a third had access to basic water and sanitation (15). This absence of water, sanitation and hygiene (WASH) clearly jeopardises birth attendants’ ability to carry-out relevant IPC practices. The reasons for this poor state of hygiene in maternity units in LMICs are multifactorial and require concerted action among a wide variety of stakeholders, from frontline care providers and cleaners, to estate managers, and to policy-makers and others ultimately accountable for maternal and newborn health and survival (16).

And what is the link with AMR? Again history shows how in high-income countries, a tolerance of poor hygiene in health institutions coincided with the growing reliance on antibiotics, which – in turn – perpetuated inappropriate use and poor drug stewardship, thus contributing to emerging resistance (17, 18). Moreover, the difficulty of distinguishing between hospital- or community-acquired infections, and the scope for risks in both directions, created ambiguity regarding where action should be targeted and a perceived need for universal precautions (19, 20). In LMICs, the comfort blanket of antibiotics for prophylactic use in clearly indicated cases, such as operative delivery, can slip seamlessly into routine use for all deliveries by healthcare workers, partly owing to their own recognition of the inadequate state of hygiene in facilities, and partly to their assumptions about the poor personal hygiene of women attending for delivery. In India and Bangladesh, for example, a recent needs assessment found that 13 of 15 maternity units, public and non-public, routinely administered antibiotics to all labouring women, irrespective of a normal or complicated delivery (21). Recent evidence (22) on the prevention of newborn infections through use of a single-dose antibiotic to all women in labour has encouraged further debate on the risks of prophylactic use as standard care where there is minimal routine monitoring of resistance and where there is still considerable room for prevention through improved hygiene. Moreover, in LMICs where delivery by caesarean section is increasing, such as in Brazil where levels have reached 57% of births (23), maternity units may have the vast majority of women inpatients receiving antibiotics – both for prophylaxis and for treatment of wound infections or other clinically-indicated reasons.

So what do we know about the magnitude of AMR on maternity units? What information is available from routine monitoring? And what is the strength of the research evidence-base? In terms of routine data, several major reports (1, 2, 24) highlight the weaknesses in the availability, representativeness, and quality of information on AMR across the globe and across the health sector, but particularly in LMICs. Maternity units thus suffer from this generalised problem of a lack of routine information. As for the magnitude of research, a crude gauge is provided by searching an established reference database. We used EMBASE, and limited the search to publications in English since 2010. To provide an indication of the maximum potential volume of research, all articles were included, regardless of the population-base or study type, and duplicates were not removed. This simple exploration revealed that the number of references from using broad search terms for AMR and hospitals was nearly 600 times greater than the number from using terms for AMR and maternity units. Among the latter, a trivial proportion of references specifically mentioned the research context being LMICs. Accepting the limitations of this crude approach, and the need for further work to conduct a robust systematic review, the conclusion is clear – there is very little published on AMR in maternity units in the very parts of the world where most births occur and where quality of care, including primary prevention of infections, is most lacking.

At the 71st session of UNGA later this month on AMR, priorities will be set. In the absence of robust evidence on the situation in maternity units, the threat from AMR and the opportunities for infection prevention and appropriate antimicrobial stewardship may simply be ignored, with serious consequences. Together with the wide variety of agencies pledging their support for global action on AMR at the UN high-level forum, such as WaterAid (25), we urge the diverse academic community – from microbiology, epidemiology, medicine, pharmacy, health services research, social science, policy analysis, and many other disciplines – to play their part in identifying and implementing a robust, action-oriented research agenda for AMR specifically targeting maternity units. Three themes are flagged to illustrate the breadth of the disciplines and innovation needed:

1. Strengthening tools, metrics, and measurement systems: Practical tools, such as standardized audit forms and simple infographics software, are needed to support the tracking of antibiotic use and to measure AMR in health service settings in LMICs. These tools must be sensitive to the limited capacity of local microbiological laboratories, including specimen transport, and to the resources needed for their use. Systems for surveillance of HCAIs – both infections captured and recorded in facilities or through community follow-up – require agreed definitions and innovations in bio-sampling and informatics in order to establish pathways for infection and the burden of resistant pathogens;
2. Developing and evaluating interventions: Health services research and innovative audits are needed on current standards of IPC in maternity units. Modalities need to be identified and tested to ensure that interventions – be these enhanced WASH, effective bed management to reduce crowding (see Fig. 2), efficient procurement of essential cleaning supplies, or better prescribing guidelines and drug stewardship – are fully integrated into broader quality improvement processes. The case for robust intervention trials of alternate drugs and regimes for the prophylaxis or treatment of maternal and newborn HCAIs should also be explored;
3. Improving the knowledge base on human behaviour around AMR: Understanding human behaviour is key to developing sustainable, effective, and affordable interventions to prevent infections and to mitigate the threat of AMR for maternity units. Strong, in-depth, social science is essential to understand and influence key preventive behaviours and practices, such as hand hygiene, infrastructural maintenance, and facility cleaning.

Women in LMICs have expressed their demand to deliver in health institutions, with more than half of births now taking place in maternity units (7). Global health action is needed to ensure that all women receive quality care (8) at birth – care that is effective, safe, and a good experience. Prevention of infections at birth, via improved WASH and IPC in maternity units is indeed better than cure – saving lives and costs, and helping to safeguard antibiotic efficacy. Combining this primary prevention with essential actions to reduce inappropriate and unnecessary antibiotic use in maternity units will ensure we can continue to save women and newborn babies in the foreseeable future.

## Paper context

The global threat from antimicrobial resistance is increasingly apparent but some at-risk groups are neglected. Sepsis deaths still occur among mothers and babies, particularly in low- and middle-income countries. The birth environment is crucial to preventing infections. Good hygiene at birth reduces risks and so impacts on the need for antibiotics and the emergence of resistance. Researchers should help strengthen the evidence-base to inform a dual strategy of infection prevention and rational drug use.
